# Supplementary material for: Content-rich biological network constructed by mining PubMed abstracts
Source: BMC Bioinformatics. 2004 Oct 8;5:147. doi: 10.1186/1471-2105-5-147 (PMC528731; doi:10.1186/1471-2105-5-147)
Supplement: Additional File 2 — The original results of the above study (non-essential files are deleted to keep the file size under the limit set by BMC bioinformatics). [file 1471-2105-5-147-S2.bz2 › chilibotAdditionalFile2/dip05/530ID9671457E10847/html/PGR_HMGB1.html]

 


 **PGR** and **HMGB1** 
  
Found 11 abstracts in PubMed, retrieved 05.  
 

 What does Google say? 
 PDF only 
| .edu only 

---

**Interactive relationship** (e.g. stimulation, inhibition, etc)

**Neutral relationship**- We have previously reported that progesterone receptor  [ **PGR** ]  requires a nuclear factor, high mobility group 1 or 2 HMG 1  [ **HMGB1** ]  2 for high affinity interaction with DNA in vitro and for full transcriptional activity in vivo.  Ref: 10503712 Steroids, 1999

**Non-interactive relationship** (e.g. studied together, co-existance, homology, etc.)

- We previously reported that the chromatin high mobility group protein 1  [ **HMGB1** ]  HMG 1  [ **HMGB1** ]  enhances the sequence specific DNA binding activity of progesterone receptor  [ **PGR** ]  PR in vitro, thus providing the first evidence that HMG 1  [ **HMGB1** ]  may have a coregulatory role in steroid receptor mediated gene transcription.  Ref: 9671457 Mol Cell Biol, 1998
